# Supplementary material for: Antibody Levels Poorly Reflect on the Frequency of Memory B Cells Generated following SARS-CoV-2, Seasonal Influenza, or EBV Infection
Source: Cells. 2022 Nov 18;11(22):3662. doi: 10.3390/cells11223662 (PMC9688940; doi:10.3390/cells11223662)
Supplement: Supplementary file 1 [file cells-11-03662-s001.zip › cells-1960390-supplementary.pdf]

Supplementary Table S1. Details of pre-COVID-19 donor cohort

Donor ID	Date of Collection	Race	Gender	Age
pre-COVID-19 Donor 1	Apr-19	Black	F	66-70
pre-COVID-19 Donor 2	Sep-18	White	F	21-25
pre-COVID-19 Donor 3	Sep-18	Hispanic/Latino	F	51-55
pre-COVID-19 Donor 4	Jun-19	White	M	51-55
pre-COVID-19 Donor 5	Jul-16	White	F	41-45
pre-COVID-19 Donor 6	Mar-17	Hispanic/Latino	M	18-20
pre-COVID-19 Donor 7	Jul-17	Hispanic/Latino	M	31-35
pre-COVID-19 Donor 8	Aug-17	Hispanic/Latino	F	36-40
pre-COVID-19 Donor 9	Sep-18	Black	M	21-25
pre-COVID-19 Donor 10	Oct-18	Hispanic/Latino	M	36-40
pre-COVID-19 Donor 11	Apr-19	Hispanic/Latino	F	56-60
pre-COVID-19 Donor 12	Aug-19	Hispanic/Latino	M	31-35
pre-COVID-19 Donor 13	Sep-19	Black	M	31-35
pre-COVID-19 Donor 14	Oct-19	Black	M	46-50
pre-COVID-19 Donor 15	Nov-11	Hispanic/Latino	M	41-45
pre-COVID-19 Donor 16	Sep-17	Black	F	26-30
pre-COVID-19 Donor 17	Dec-17	White	M	56-60
pre-COVID-19 Donor 18	Mar-18	Hispanic/Latino	M	31-35
pre-COVID-19 Donor 19	Mar-18	Black	F	56-60
pre-COVID-19 Donor 20	May-18	Black	M	46-50
pre-COVID-19 Donor 21	May-18	Hispanic/Latino	F	26-30
pre-COVID-19 Donor 22	Jul-17	Hispanic/Latino	M	41-45
pre-COVID-19 Donor 23	Jul-18	Hispanic/Latino	M	41-45
pre-COVID-19 Donor 24	Aug-18	Hispanic/Latino	M	26-30
pre-COVID-19 Donor 25	Aug-18	Asian	M	36-40
pre-COVID-19 Donor 26	Sep-18	Black	M	26-30
pre-COVID-19 Donor 27	Oct-18	White	M	41-45
pre-COVID-19 Donor 28	Oct-18	Hispanic/Latino	M	26-30
pre-COVID-19 Donor 29	Oct-18	Black	F	26-30
pre-COVID-19 Donor 30	Oct-18	Asian (Filipino)	M	26-30
pre-COVID-19 Donor 31	Oct-18	Pacific Islander	M	36-40
pre-COVID-19 Donor 32	Oct-18	White	F	26-30
pre-COVID-19 Donor 33	Apr-19	Black	M	51-55
pre-COVID-19 Donor 34	Apr-19	Hispanic/Latino	M	26-30
pre-COVID-19 Donor 35	Apr-19	Hispanic/Latino	M	36-40
pre-COVID-19 Donor 36	May-19	Hispanic/Latino	F	46-50
pre-COVID-19 Donor 37	May-19	Hispanic/Latino	M	56-60
pre-COVID-19 Donor 38	May-19	Hispanic/Latino	M	56-60
pre-COVID-19 Donor 39	May-19	Hispanic/Latino	M	26-30
pre-COVID-19 Donor 40	Jun-19	White	M	61-65
pre-COVID-19 Donor 41	Jun-19	Hispanic/Latino	F	51-55
pre-COVID-19 Donor 42	Jun-19	Hispanic/Latino	M	31-35
pre-COVID-19 Donor 43	Jun-19	White	M	41-45
pre-COVID-19 Donor 44	Jul-19	White	M	51-55
pre-COVID-19 Donor 45	Jul-19	Hispanic/Latino	M	21-25
pre-COVID-19 Donor 46	Jul-19	White	M	26-30
pre-COVID-19 Donor 47	Jul-19	White	M	36-40
pre-COVID-19 Donor 48	Jul-19	Hispanic/Latino	M	41-45
pre-COVID-19 Donor 49	Aug-19	Black	M	31-35
pre-COVID-19 Donor 50	Sep-19	Hispanic/Latino	M	26-30
pre-COVID-19 Donor 51	Sep-19	Hispanic/Latino	F	36-40
pre-COVID-19 Donor 52	Oct-19	Black/Hispanic/Latino	M	26-30
pre-COVID-19 Donor 53	Oct-19	White	M	31-35
pre-COVID-19 Donor 54	Oct-19	White	F	61-65

Supplementary Table S2. Details of COVID-19 donor cohort

Donor ID	Date of sample collection	Length of convalescence (days)*	Race	Gender	Age
COVID-19 Donor 1	Oct-20	187	White	F	46-50
COVID-19 Donor 2	Sep-20	155	White	F	46-50
COVID-19 Donor 3	Oct-20	130	White	F	56-60
COVID-19 Donor 4	Aug-20	110	White	F	36-40
COVID-19 Donor 5	Sep-20	176	White	F	56-60
COVID-19 Donor 6	Apr-20	25	unknown	M	66-70
COVID-19 Donor 7	Apr-20	26	unknown	M	61-65
COVID-19 Donor 8	Apr-20	15	unknown	F	21-25
COVID-19 Donor 9	Apr-20	19	unknown	M	36-40
COVID-19 Donor 10	Apr-20	37	unknown	M	36-40
COVID-19 Donor 11	May-20	38	White	F	41-45
COVID-19 Donor 12	May-20	20	White	M	21-25
COVID-19 Donor 13	May-20	unknown	Black	F	51-55
COVID-19 Donor 14	May-20	14	Black	M	46-50
COVID-19 Donor 15	May-20	14	Black	F	18-20
COVID-19 Donor 16	May-20	17	Black	F	21-25
COVID-19 Donor 17	Jun-20	34	White	F	26-30
COVID-19 Donor 18	Jul-20	unknown	White	F	51-55
COVID-19 Donor 19	Jul-20	34	White	F	21-25
COVID-19 Donor 20	Jul-20	95	White	F	51-55
COVID-19 Donor 21	Jul-20	24	Black	M	31-35
COVID-19 Donor 22	Jul-20	20	Hispanic/Latino	F	21-25
COVID-19 Donor 23	Jul-20	32	White	M	26-30
COVID-19 Donor 24	Aug-20	unknown	White	F	31-35
COVID-19 Donor 25	Oct-20	174	White	F	41-45

* number of days between collection of PBMC and positive SARS-CoV-2 PCR result

Supplementary Table S3. Antibody-secreting cell (ASC) reactivity against SARS-CoV-2 and third party antigens

Donor ID	Total IgG†	6XHis peptide#	NCAP* (SARS-CoV-2)	S1 subunit# (SARS-CoV-2)	CA/09 rHA# (H1N1)	TX/12 rHA# (H3N2)	Phuket/13 rHA# (FluB)	EBNA1# (EBV)
pre-COVID-19 Donor 5	63	<5	<5	<5	43	91	14	5
pre-COVID-19 Donor 6	140	<5	<5	<5	134	20	<5	<5
pre-COVID-19 Donor 7	97	<5	<5	<5	95	24	8	<5
pre-COVID-19 Donor 8	103	<5	<5	<5	9	25	10	27
pre-COVID-19 Donor 9	74	<5	<5	<5	15	20	36	21
pre-COVID-19 Donor 10	134	<5	<5	<5	49	56	162	77
pre-COVID-19 Donor 11	99	<5	<5	<5	93	60	52	<5
pre-COVID-19 Donor 12	65	<5	<5	<5	86	36	8	6
pre-COVID-19 Donor 13	20	<5	<5	<5	5	6	<5	<5
pre-COVID-19 Donor 14	96	<5	<5	<5	274	167	233	48
COVID-19 Donor 2	86	<5	124	100	61	66	43	19
COVID-19 Donor 5	55	<5	26	63	37	11	34	11

† Mean spot-forming unit (SFU) counts at 937 PBMC/well

#Mean SFU counts at 3 x 10^5 PBMC/well

Supplementary Table S4. Association of host factors and discordance between antibody levels and SARS-CoV-2 antigen-reactive B_{mem} frequencies

Antigen	Subgroup	Subgroup size	R^2	p-value[#]
SARS-CoV-2 Spike (S1)	Sex (Female)	17	0.208	0.0659
	Sex (Male)	8	0.451	0.0683
	Age (<35 years)	10	0.292	0.1071
	Age (36-55 years)	11	0.064	0.4545
	Age (56+ years)	4	0.64	0.1998
	Race/Ethnicity (White)	14	0.285	0.0492
	Race/Ethnicity (Non-white)	6	0.345	0.22
	Time since infection (<45 days)	15	0.382	0.0141
	Time since infection (45+ days)	7	0.105	0.4792
SARS-CoV-2 NCAP	Sex (Female)	17	0.023	0.5589
	Sex (Male)	8	0.284	0.1743
	Age (<35)	10	0.223	0.1683
	Age (36-55)	11	0.148	0.243
	Age (56+)	4	0.532	0.2705
	Race/Ethnicity (White)	14	0.002	0.8714
	Race/Ethnicity (Non-white)	6	0.396	0.1806
	Time since infection (<45 days)	15	0.263	0.0507
	Time since infection (45+ days)	7	0.066	0.5773

[#]p-values less than 0.05 are denoted by red text

Table S5. Association of host factors and discordance between antibody levels and third party antigen-reactive B_{mem} frequencies

Antigen	Subgroup	Subgroup size	R^2	p-value[#]
A/California/04/2007 (CA/09) rHA	Sex (Female)	35	0.0387	0.2573
	Sex (Male)	44	0.3146	<0.0001
	Age (<35 years)	35	0.1755	0.0123
	Age (36-55 years)	32	0.2564	0.0031
	Age (56+ years)	12	0.1008	0.3147
	Race/Ethnicity (White)	27	0.0495	0.2645
	Race/Ethnicity (Non-white)	47	0.2934	<0.0001
A/Texas/50/2012 (TX/12) rHA	Sex (Female)	35	0.1502	0.0214
	Sex (Male)	44	0.0223	0.3329
	Age (<35 years)	35	0.2229	0.0042
	Age (36-55 years)	32	0.0927	0.0903
	Age (56+ years)	12	<0.0001	0.9934
	Race/Ethnicity (White)	27	0.0505	0.2598
	Race/Ethnicity (Non-white)	47	0.1809	0.0029
B/Phuket/3073/2013 (Phuket/13) rHA	Sex (Female)	35	0.0658	0.1369
	Sex (Male)	44	0.1226	0.0198
	Age (<35 years)	35	0.078	0.1043
	Age (36-55 years)	32	0.101	0.0764
	Age (56+ years)	12	0.127	0.2556
	Race/Ethnicity (White)	27	0.1292	0.0656
	Race/Ethnicity (Non-white)	47	0.1143	0.0201
EBNA1 (EBV)	Sex (Female)	35	0.2457	0.0025
	Sex (Male)	44	0.2026	0.0022
	Age (<35 years)	35	0.1727	0.013
	Age (36-55 years)	32	0.2982	0.0012
	Age (56+ years)	12	0.0051	0.8258
	Race/Ethnicity (White)	27	0.2549	0.0072
	Race/Ethnicity (Non-white)	47	0.3768	<0.0001

[#]p-values less than 0.05 are denoted by red text

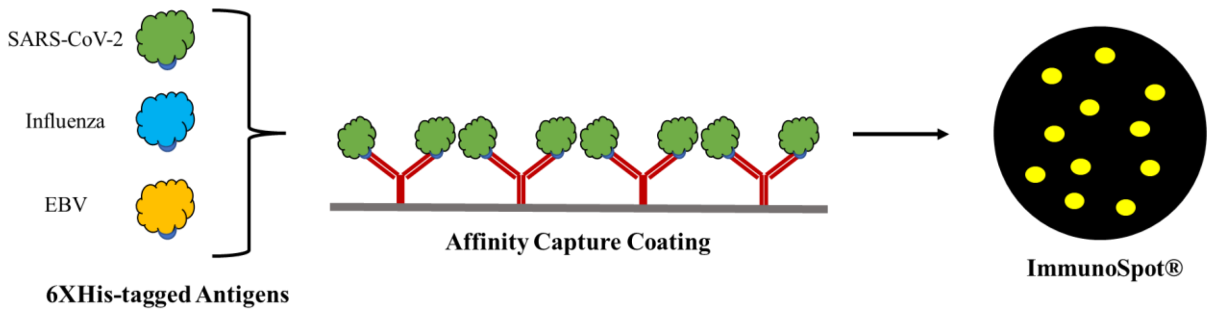

Supplementary Figure S1: Schematic representation of affinity capture coating of ELISPOT/FluoroSpot membranes. The approach shown is suited for recombinant antigens that are His-tagged, such as the SARS-CoV-2, influenza, or EBV antigens used in this study. The PVDF membrane on the bottom of the assay plate is first coated with an anti-His antibody, that in a subsequent coating step, binds the His-tag of the recombinant antigen. The next step of the ELISPOT/FluoroSpot assay (not shown) is the addition of PBMC containing antibody-secreting cells (ASC). During this cell culture period, while all ASC (irrespective of their antigen specificity) will secrete antibody, only the immunoglobulin released by antigen-reactive ASC will be captured on the membrane in close proximity to generate a antibody secretory foot-print. The resulting plate-bound secretory antibody foot-prints are then detected in the final steps of the assay, and the spot-forming units (SFU) are visualized and counted.

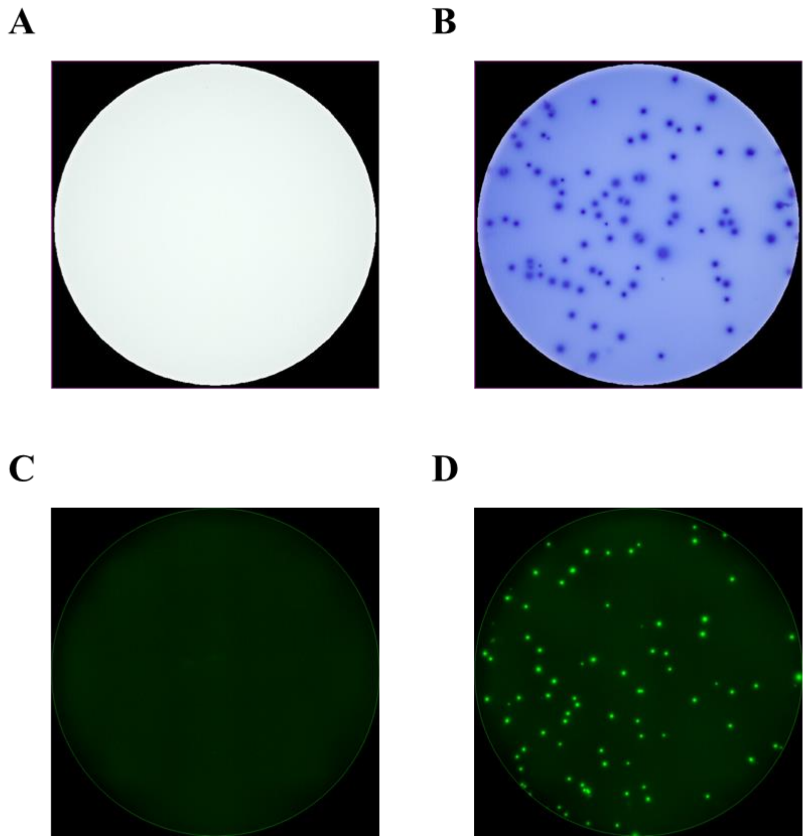

Supplementary Figure S2: Formation of secretory foot-prints requires coating of PVDF membranes with capture antibody. Murine B cell hybridoma cells were incubated for 6hr in either ELISPOT (A and B) or FluoroSpot (C and D) assay plates and the resulting antibody-derived secretory foot-prints were revealed. In contrast to wells that were coated with capture antibody, subsequently blocked prior to input of hybridoma cells, and yielded well-defined secretory foot-prints (B and D), wells that were treated analogously but did not receive capture antibody failed to yield detectable secretory foot-prints (A and C). **Note:** While FluoroSpot data are presented in the main text, we also included ELISPOT results owing to the increased sensitivity afforded using this technique through the enzymatic amplification step. Collectively, these data exclude the possibility that secreted Ig can yield a detectable secretory foot-print on the PVDF membrane in the absence of dedicated capture reagents.

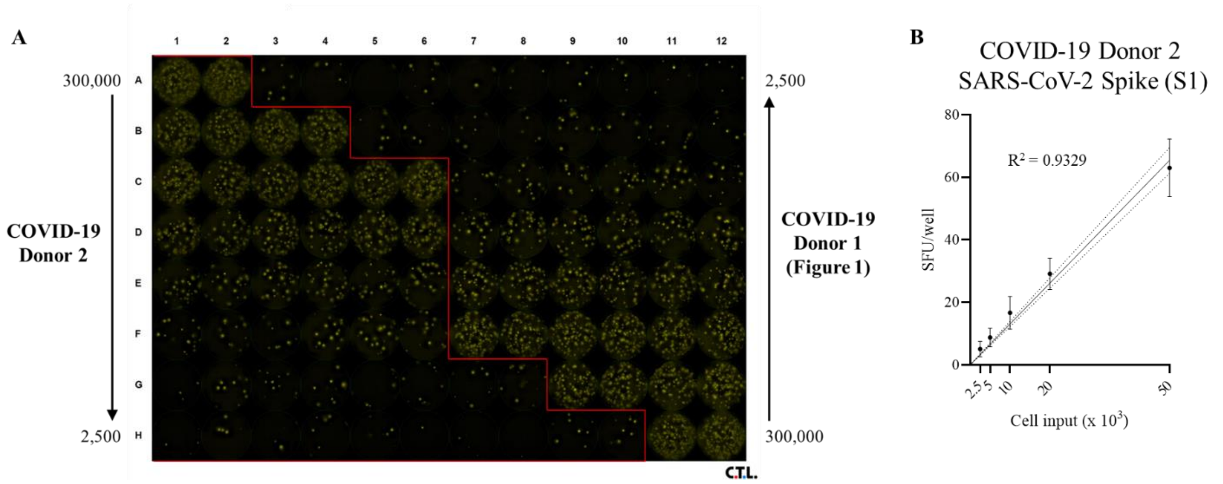

Supplementary Figure S3. Accurate measurement of SARS-CoV-2 Spike (S1) antigen-reactive memory B cell frequencies illustrated using two convalescent COVID-19 donors. (A) PBMC (that were pre-stimulated for 5 days *in vitro* to transition resting B cells, irrespective of their specificity, to antibody-secreting cells (ASC) as detailed in *Materials and Methods*) were plated in decreasing cell inputs, starting at 3 x 10^5, progressing for COVID-19 Donor 2 from row A to H, and for COVID-19 Donor 1 from row H to A, in increasing numbers of replicate wells, as shown. Red line depicts the separation of wells seeded with cells from the two respective donors. SARS-CoV-2 Spike (S1)-reactive IgG^+ ASC were then detected as described in Figure S1 and *Materials and Methods*. (B) Linearity between cell input and spot-forming unit (SFU) counts are seen at less than 5 x 10^4 PBMC/well for COVID-19 Donor 2. Means and SD of the replicate wells are shown with regression calculated, which establishes the frequency of SARS-CoV-2 Spike (S1) antigen-reactive IgG^+ B cells in COVID-19 Donor 2 at 163 SFU/10^5 PBMC. The SFU counts for COVID-19 Donor 1 are shown in Figures 1D and 1E.

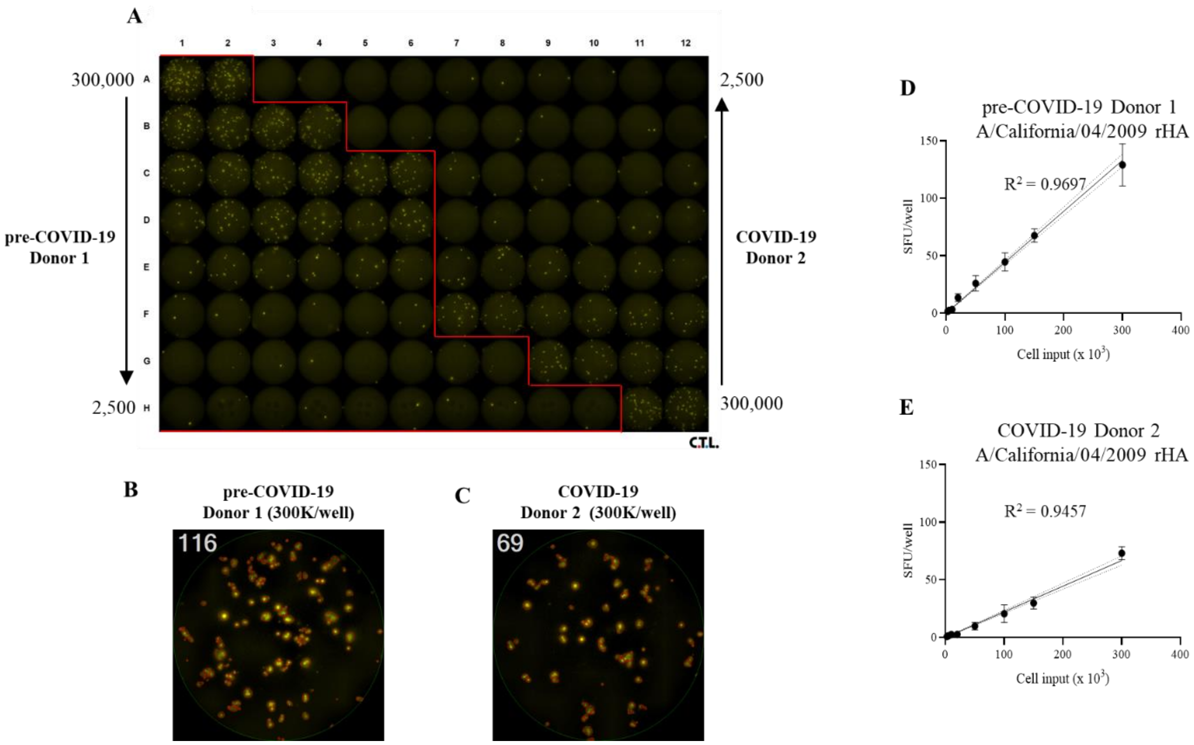

Supplementary Figure S4. Accurate measurement of seasonal influenza A (A/California/04/2009, CA/09) recombinant hemagglutinin (rHA)-reactive memory B cell frequencies illustrated using PBMC donors. (A) PBMC from pre-COVID-19 Donor 1 and COVID-19 Donor 2 were pre-stimulated, as detailed in *Materials and Methods*, before seeding into a FluoroSpot assay following the same plate layout as described in Figure S3, and the CA/09 rHA-reactive IgG^+ ASC were detected. (B) and (C) shows original images for both donors at 3×10^5 PBMC/well; (D) and (E) shows the SFU counts versusthe cell input for pre-COVID-19 Donor 1 and COVID-19 Donor 2, respectively. In other details, the legend for Figure S3 applies. Note, because of the relative low frequency of CA/09 rHA-reactive ASC, the linear range in both donors extended to 3×10^5 PBMC/well. Based on linear regression, the frequency of CA/09 rHA-reactive IgG^+ B cells in pre-COVID-19 Donor 1 and COVID-19 Donor 2 was established at 50 and 23 SFU/10^5 PBMC, respectively.

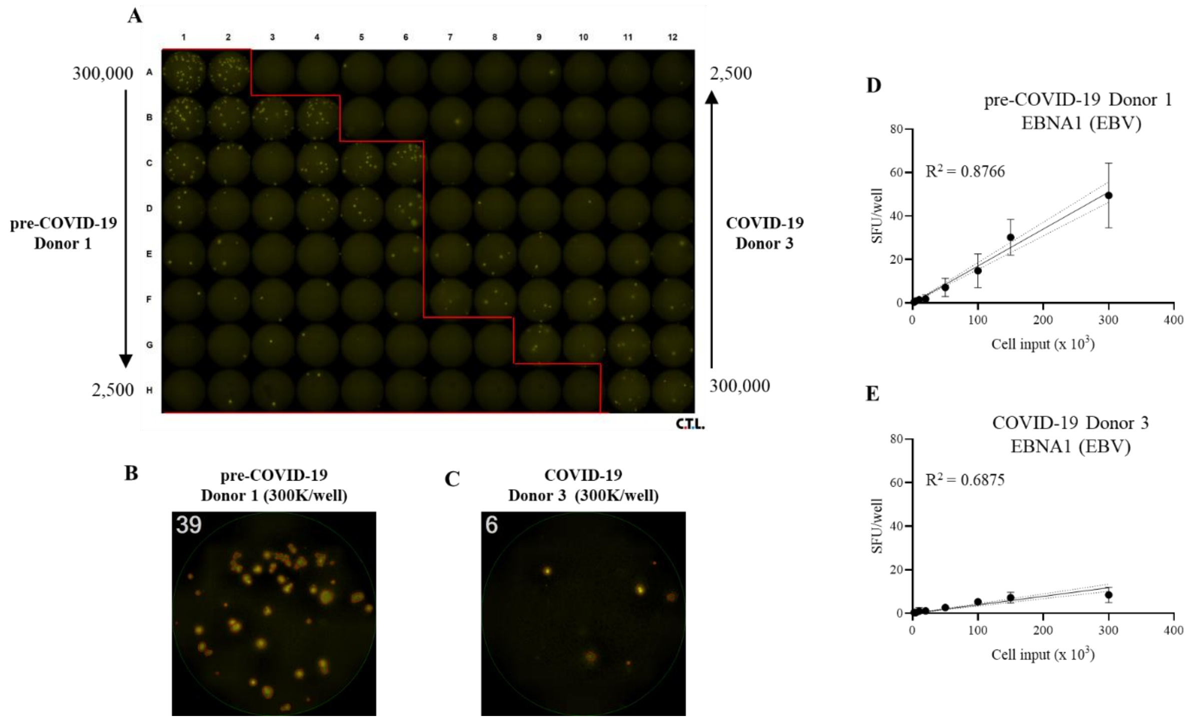

Supplementary Figure S5. Accurate measurement of EBV EBNA1-reactive memory B cell frequencies illustrated using PBMC donors. (A) PBMC from pre-COVID-19 Donor 1 and COVID-19 Donor 3 were pre-stimulated, as detailed in *Materials and Methods*, before seeding into a FluoroSpot assay following the same plate layout as described in Figure S3, and the EBNA1-reactive IgG^+ ASC detected. (B) and (C) shows original images for both donors at 3×10^5 PBMC/well; (D) and (E) shows the SFU counts versus the cell input for pre-COVID-19 Donor 1 and COVID-19 Donor 3, respectively. In other details, the legend for Figure S3 applies. Note, because of the relative low frequency of EBNA1-reactive ASC, the linear range in both donors extended to 3×10^5 PBMC/well. Based on linear regression, the frequency of EBNA1-reactive IgG^+ B cells in pre-COVID-19 Donor 1 and COVID-19 Donor 3 was established at 16 and 5 SFU/10^5 PBMC, respectively.

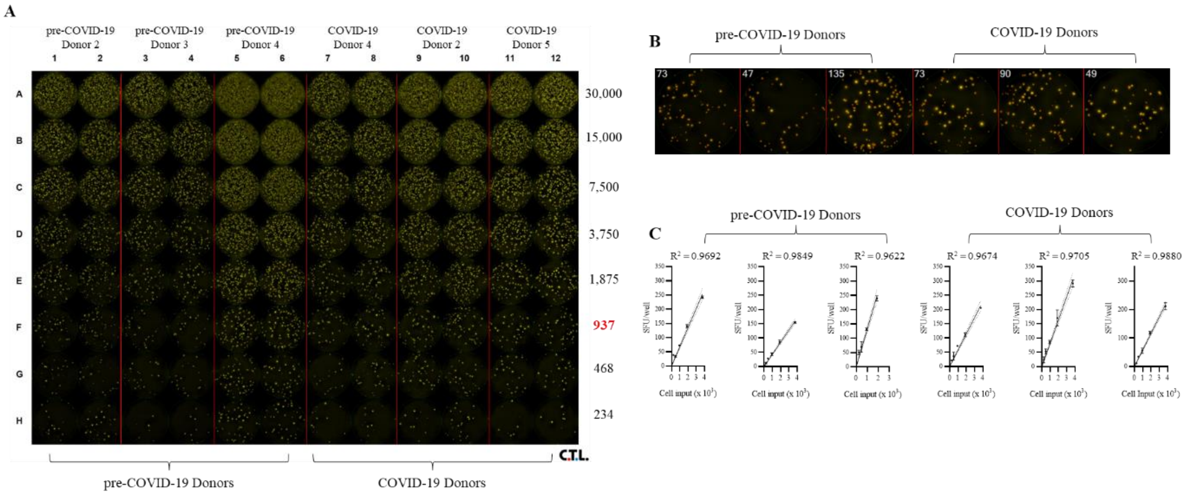

Supplementary Figure S6. Accurate measurement of total IgG^+ ASC frequencies illustrated. (A) Overview image of an ImmunoSpot® assay plate showing test results for polyclonally-stimulated PBMC from three pre-COVID-19 era donors (columns 1-6) and three convalescent COVID-19 donors (columns 7-12). Each donors' cells were plated in duplicate at a starting input of 3 x 10^4 PBMC/well and were serially diluted two-fold down the plate. The approximate cell inputs for each row are specificied on the right. Total IgG^+ ASC, irrespective of their antigen reactivity, were then detected as detailed in *Materials and Methods.* (B) Enlarged well images for all six donors at the 973 PBMC/well dilution step, with the SFU counts shown in the upper left corner of each well. (C) Linearity of cell numbers plated versus SFU counts in the range tested. Based on linear regression, the frequency of IgG^+ ASC in these donors was established (from left to right) as 7,484 (pre-COVID-19 Donor 2), 4,444 (pre-COVID-19 Donor 3), 13,875 (pre-COVID-19 Donor 4), 6,690 (COVID-19 Donor 4), 9,866 (COVID-19 Donor 2) and 5,981 IgG^+ ASC/10^5 PBMC, respectively.

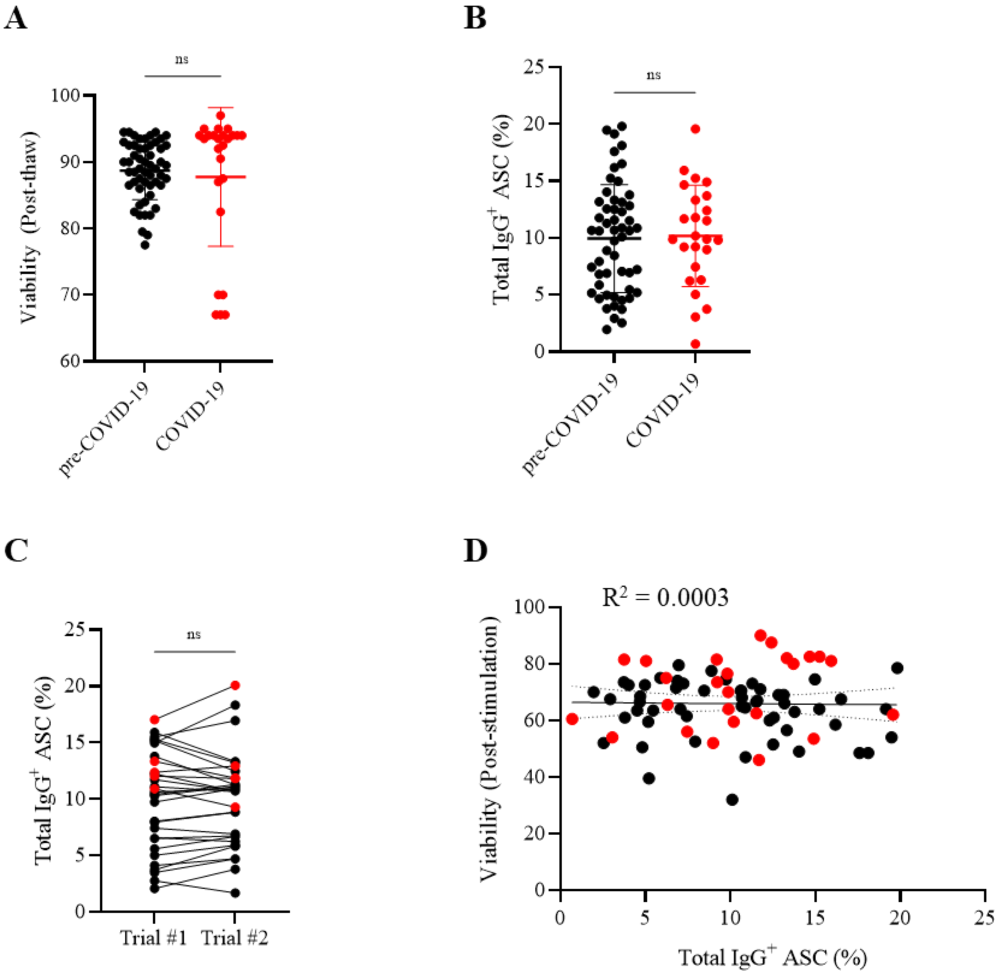

Supplementary Figure S7. Similar viability and frequency of total IgG+ ASC in subjects whose PBMC were cryopreserved in the pre-COVID-19 era (in black) versus convalescent COVID-19 PBMC donors (in red). (A) Viability of PBMC established upon thawing the cryopreserved cells. Viability was defined as the percentage of cells staining with the acridine orange vital dye versus dead cells that were propidium iodide positive. Mean and SD is shown for both cohorts. Of note, the five COVID-19 donors displaying reduced viability upon thawing were obtained from a single source (Oklahoma Blood Institute). (B) Total IgG+ ASC frequencies expressed as a percentage of viable PBMC following five days of *in vitro* stimulation (detailed in *Materials ands Methods)*. Means and SD are shown for both cohorts. (C) Reproducibility of total IgG+ ASC frequency between independent experimental trials. A line connects the data points obtained for each donor. (D) Total IgG+ ASC frequency as a function of PBMC viability (determined as in A) on day 5 prior to seeding the cells into FluoroSpot assays. Each donor's cells are represented by a dot and the black (pre-COVID-19) or red (COVID-19) color code defines their SARS-CoV-2 exposure status.

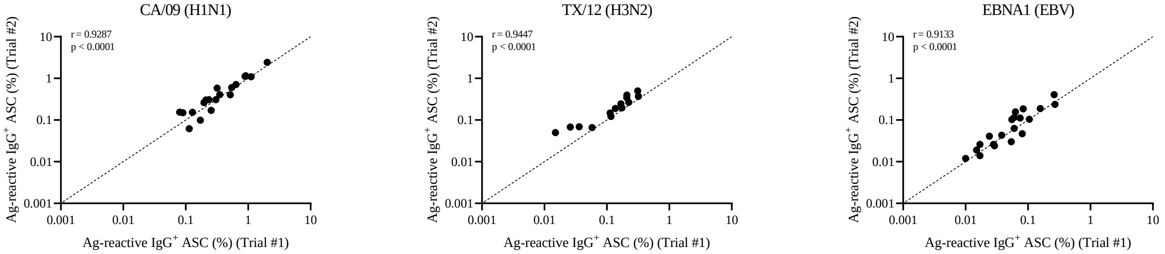

Supplementary Figure S8. Reproducibility of antigen (Ag)-reactive ASC frequency measurements across independent experiments. PBMC from each subject (represented by a dot) were tested in two independent experiments and the established frequency of Ag-reactive IgG+ ASC in Trial #1 is plotted on the x-axis and the Ag-reactive IgG+ ASC frequency in Trial #2 is plotted on the y-axis. The dotted line (line of identity) indicates perfect reproducibility. Results are shown for detection of IgG+ ASC with reactivity against (A) A/California/04/09 (CA/09) recombinant hemagglutinin (rHA), (B) A/Texas/50/2012 (TX/12) rHA or (C) EBV EBNA1 proteins, respectively.

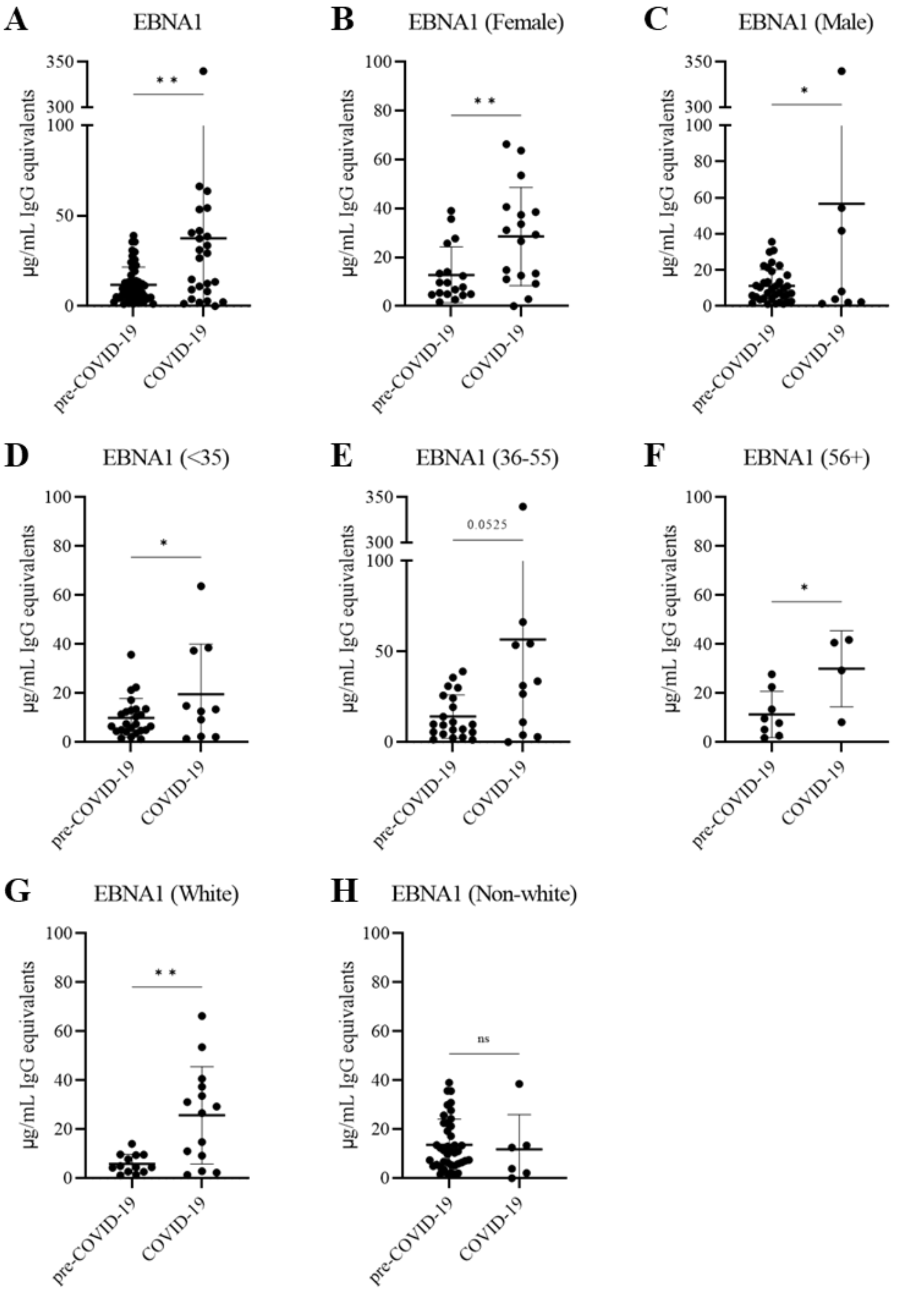

Supplementary Figure S9: Increased plasma IgG reactivity against EBNA1 in COVID-19 donors. (A) EBNA1-reactive circulating IgG antibody levels in pre-COVID-19 and COVID-19 donors were measured by ELISA (detailed in *Materials and Methods*) alongside an IgG reference standard that enabled interpolation of EBNA1-reactive IgG levels as "μg/mL equivalents". Donors in the pre-COVID-19 and COVID-19 cohorts were further subcategorizes according to (B and C) sex, (D-F) age, or (G and H) race/ethnicity. Statistical significance was determined using an unpaired Student's t-test. *$p<0.05$, **$p<0.01$
